# Accounting for direct and indirect cumulative effects of anthropogenic pressures on salmon- and herring-linked land and ocean ecosystems

**DOI:** 10.1098/rstb.2021.0130

**Published:** 2022-07-04

**Authors:** Vivitskaia J. D. Tulloch, Megan S. Adams, Tara G. Martin, Ayesha I. T. Tulloch, Rebecca Martone, Stephanie Avery-Gomm, Cathryn C. Murray

**Affiliations:** ^1^ Conservation Decisions Laboratory, Department of Forest and Conservation Science, Faculty of Forestry, University of British Columbia, Vancouver, British Columbia, Canada; ^2^ School of Life and Environmental Sciences, University of Sydney, Sydney, New South Wales, Australia; ^3^ Ministry of Forests, Lands, Natural Resource Operations, and Rural Development, Coast Region, Province of British Columbia, Victoria, British Columbia, Canada; ^4^ Environment and Climate Change Canada, Science and Technology Branch, Ottawa, Ontario, Canada; ^5^ Fisheries and Oceans Canada, Institute of Ocean Sciences, Sidney, British Columbia, Canada

**Keywords:** foundation species, cumulative effects, cumulative impact, cross-realm, food web, indirect effects

## Abstract

Salmon and herring support both land and ocean predators and are critical to ecosystem resilience. Their linkages across land and sea realms make them highly susceptible to human activities, which can have flow-on effects up the food web. We quantify and compare the potential cumulative effects of human-driven pressures on interdependent species in salmon- and herring-linked ecosystems of western Canada using a risk assessment methodology. Adding indirect risks resulted in 68% greater total risks for land species than for direct risk alone, versus 15% for marine species. Inclusion of climate change pressures resulted in the greatest change in risk for low trophic marine species and habitats (greater than 25% increase). Forestry-related pressures accounted for the highest risk to all species and projected management of these pressures resulted in a total reduction of risk across all ecosystem components that was more than 14% greater than management of fisheries pressures. Ignoring land food web linkages and pressures underestimated cumulative risk by more than 40% for salmon and herring. This simple framework can be used to evaluate potential risk of existing human uses and future change to inform immediate management of linked land-sea ecosystems and help species avoid the ‘death by a thousand cuts'.

This article is part of the theme issue ‘Nurturing resilient marine ecosystems’.

## 1. Introduction

Foundation species play pivotal ecological roles in supporting community structure and ecosystem functioning from the bottom up, are essential to ecosystem integrity and resilience [1], and often have important cultural and economic value [2]. Foundation species are generally abundant enough to directly and indirectly connect to many more species than other species in an ecological network [1], particularly those using coastal environments that typically provide vital food sources for both land and ocean predators [3]. Coastal environments are highly threatened by exposure to both ocean- and land-based human pressures [4,5], as well as future climate change [6]. Ongoing and concurrent pressures can affect current and future resilience and responses of coastal foundation species to change, and can flow on to have disproportionate impacts on key ecologically linked species [7]. These flow-on effects may jeopardize conservation and management of species and compromise ecosystem processes.

In coastal environments in western North America, the foundation species Pacific salmon (*Oncorhynchus* spp.) and Pacific herring (*Clupea pallasi*) provide critical prey to species in surrounding coastal ecosystems [8], a process which is threatened by overlapping and intersecting pressures of climate change, habitat degradation, and commercial exploitation in land and marine environments. Marine mammals depend directly on Pacific salmon for food [9], while land mammals rely on adult salmon as a key food source upon their return to freshwater rivers [10]. Migrating juvenile salmon or spawned-out adult salmon carcasses are important annual prey sources for a broad array of species in freshwater and terrestrial systems [11,12]. The salmon predator–prey system helps distribute marine nutrients from salmon flesh into land ecosystems [8,13] through direct carcasses deposition or scat and urine deposits from predators [14], thus further linking marine and land ecosystems across the range of salmon distribution. Pacific herring is another example of a foundational species which support a vast foodweb from open ocean to nearshore ecosystems. Juvenile and adult herring are critical prey for open ocean and nearshore predators [15], while shoreline spawning events also support an array of predators and scavengers through pulsed aggregations of fishes and lipid-rich eggs [16] that benefit seabirds, landbirds, marine mammals, land mammals and intertidal invertebrates [3,16].

Previous research on salmon- and herring-linked ecosystems has identified the importance of ecosystem-based management that accounts for the myriad interactions between species and systems, and accounts for their complex cross-realm life history (e.g. [17,18]). Despite this, western management agencies operate in single realm jurisdictions (e.g. in Canada, federal agencies manage marine environments while provincial agencies manage terrestrial ones). As such, salmon, herring and other species at the land-sea interface often ‘slip through the cracks’ created by multi-agency jurisdiction over marine and terrestrial environmental management [19,20]. By contrast, a resurgence in Indigenous-led governance in Canada is founded on an integrated cross-realm management approach (e.g. [21]).

Given the high exposure of coastal environments to multiple human activities, a sound understanding of the cumulative direct and indirect impacts of current and future pressures on coastal foundation and linked species and overall ecosystem resilience is urgently needed to inform effective ecosystem-based management [22,23]. Often, threat management begins with a risk assessment that rapidly quantifies differences in the potential direct impacts of one or many human activities on species or habitats at an ecosystem level [24–26]. In such assessments, high risk is assumed to have negative implications for population dynamics and community structure. However, our ability to understand the risks of cumulative pressures from multiple human activities is challenged by the often complex interactions between key ecologically-linked species, particularly when impacts are indirect, as is the case when impacts are mediated by changes in habitats, prey or predators. Indirect impacts of human activities can significantly alter the structure of marine communities [27] and drive species declines in predators through changes in their prey [28]. These indirect effects can be difficult to isolate and disentangle from other direct processes, and are not often accounted for in risk assessment (but see [29]). An understanding of the flow-on impacts of both current and future pressures acting in the ecologically linked land and marine realms is necessary to guide effective management of foundation species, like Pacific salmon and herring, and those species that are dependent on them. Effective management would bolster ecological resilience and avoid the loss of essential foundation species to ‘death by a thousand cuts'.

Here, we expand upon previous risk assessment methodology [29] for evaluating both direct and indirect cumulative effects of human activities in marine environments, which allows decision-makers to explore how risk propagates through food webs, by including ecosystem components across land, freshwater and marine realms to offer a fully integrated land-sea assessment. We explore the effectiveness of single realms versus cross-realm assessments by comparing the combined risks for ecosystem components in separate land and marine assessments, where links to species and pressures in different realms are ignored, versus the cross-realm assessment. We demonstrate the approach using a case study of salmon- and herring-linked ecosystem components in an area known as the Great Bear Rainforest, British Columbia, Canada. We conduct sensitivity analyses of the importance of prey items to predator diet, and evaluate changes under future climate change and management of pressures across the land and sea.

## 2. Methods

### (a) Case study region

The coastal temperate rainforest and adjacent marine environment of the territories of the Haíɫzaqv, Kitasoo Xai'xais, Nuxalk and Wuikinuxv First Nations, or what is now known as the Central Coast region of British Columbia (BC), Canada, is the focus of this risk assessment. This culturally and ecologically important region, also popularly referred to as the Great Bear Rainforest, is characterized by glaciated fjords and a complex archipelago of exposed and protected shoreline and marine environments [30]. Pacific salmon and herring provide key foods for ecological and human communities across the Central Coast. Salmon and herring have provided key foods for local Indigenous people for millennia [31,32], and more recently supported large-scale commercial fisheries prior to widespread declines in abundance of both species. Areas of the Central Coast shorelines still support herring spawn events, and five species of Pacific salmon still spawn in many small to large rivers across the region, albeit in substantially lower abundance than historically [33]. The Central Coast is largely un-roaded and undisturbed by large industrial development, though marine shipping, commercial fishing, finfish aquaculture and commercial forestry are among chronic impacts on the landscape since colonization. Tourism is also a growing economic contributor to the region.

### (b) Land-sea conceptual model

**Figure 1.**
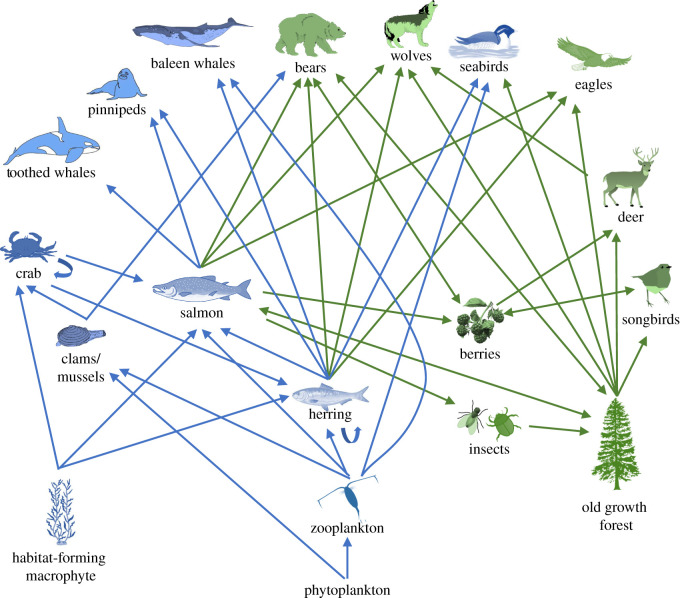
Conceptual risk pathways (food and biogenic habitat) for all the trophic groups of the ecosystem components considered, with land ecosystem components and pathways in green, and marine ecosystem components and pathways identified by [29] in blue. Pathways directionality is shown as one-way or two-way interaction arrows.

We developed a conceptual model of primary species and habitats (hereafter ‘ecosystem components’) relevant to salmon- and herring-linked ecosystems of the Great Bear Rainforest across the land and sea. A marine pathways of effects model developed for the North Pacific coast in BC [29] was used to identify the primary marine species within the salmon-linked food web to be included in the model (table 1). These linkage pathways were based on food web interactions and habitat components on which species depend (figure 1). We used literature reviews and expert consultation with local and scientific knowledge holders and managers to identify land species and habitats with key ecological-linkages to Pacific salmon and herring in the Central Coast region, and combined these with the marine components to create the land and marine conceptual model of pathways for all identified ecosystem components (table 1, figure 1).

**Table 1.** Ecosystem components assessed in the qualitative risk assessment; see the electronic supplementary material, table S2 for literature used to support linkages.

trophic group	ecosystem component	scientific name	realm	bottom-up link from foundation species (salmon or herring)	no. linkages to supporting species	no. direct linkages to consumers
phytoplankton	phytoplankton		marine		0	2
zooplankton	zooplankton		marine		1	5
habitat-forming macrophytes	seagrasses	*Zostera* spp.	marine		0	3
	kelp					
low mobility invertebrates	clams		marine		2	2
mobile benthic invertebrates	Dungeness crab	*Cancer magister*	marine		3	2
forage fishes	Pacific herring	*Clupea pallasi*	marine		2	6
anadromous fishes	Pacific salmon	*Onchorhynchus* spp.	marine/land	yes	4	8
baleen whales	humpback whale	*Megaptera novaeangliae*	marine	yes	2	0
toothed whales	resident orca	*Orcinus orca*	marine	yes	1	0
pinnipeds	Steller sea lion	*Eumetopias jubatus*	marine	yes	2	0
seabirds	marbled murrelet	*Brachyramphus marmoratus*	marine/land	yes	2	0
habitat-forming forest	old growth forest		land	yes	3	6
salmon-dependent insects	blow flies	*Calliphoridae*	land	yes	1	1
	burying beetle	*Nicrophorus investigator*				
berries	salmonberry	*Rubus spectabilis*	land		1	3
bears	grizzly bear	*Ursus arctos*	land	yes	5	1
	black bear	*Ursus americanus*				
wolves	grey wolf	*Canis lupis*	land	yes	4	0
eagles	bald eagle	*Haliaeetus leucocephalus*	land	yes	3	0
songbirds	varied thrush	*Ixoreus naevius*	land		1	2
deer	white-tailed deer	*Odocoileus virignianus*	land		2	1

### (c) Assessing human activities

Marine- and land-based human activities were evaluated for relevance to the ecosystem components in the Central Coast region. We selected a comprehensive list based on their potential consequences for the ecosystem components, their current occurrence in the region, or their expected future occurrence (electronic supplementary material, table S1). Marine-based pressures included fisheries, finfish aquaculture, log sorting and transporting, marine tourism, oil and gas exploration, wind and hydro energy, vessel use, long-range pressures such as pollutants and debris and ports, marinas and harbours (activities *n* = 8, pressures *n* = 41, electronic supplementary material, table S1). Land-based pressures included forestry, human land conversion and settlements, land tourism, mining and energy exploration and production, transport and population centres (activities *n* = 7, pressures *n* = 26). We also considered freshwater pressures derived from changing land activities (activities *n* = 5, pressures *n* = 9, electronic supplementary material, table S1). In order to fully examine cumulative impacts for the region, seven global pressures owing to climate change were examined in a futures scenario (electronic supplementary material, table S1).

### (d) Qualitative risk assessment

We used an existing risk assessment scoring framework [29] to explore combined risk of multiple human pressures to the ecosystem components. The relative direct risk of pressures to an ecosystem component was calculated as the product of the *exposure* to a pressure and the *consequence* or the sensitivity of that population to that same pressure.

The relative risk to each ecosystem component by pressure was calculated as follows:

$${\mathrm{risk}}_{ij}={\mathrm{exposure}}_{i}\times {\mathrm{consequence}}_{ij}^{2},$$

where *risk_ij_* to each ecosystem component *j* was the product of the *exposure*_*i*_ of pressure *i* (one of the land, freshwater, or marine-based human pressures, electronic supplementary material, table S1) and the *consequence_ij_* of each ecosystem component *j* being exposed to pressure *j.* Qualitative scoring of the risk variables used the same scoring methodology defined by [24] (electronic supplementary material, table S3). *Exposure*_*i*_ of each pressure *i* was the product of three variables: *temporal scale_i_* (*TS*, scored between 0 and 4), *spatial scale_i_* (*SS*, scored between 0 and 3), and *load_i_* (*L*, scored between 0 and 3, see the electronic supplementary material, table S3 for definitions of each term):

$${\mathrm{exposure}}_{i}=TS_{i}\times SS_{i}\times L_{i}.$$

*Consequence* was scored from 1 to 6 for the ecosystem component at the scale of individual pressures, and was subsequently squared to allow for equal weighting/contribution of *exposure* and *consequence* to overall risk. We used a combination of expert review and spatial data to refine each of the large-scale exposure (TS, SS and L) variable scores for BC from [29] to represent the pressures and ecosystem components of the Central Coast region (see the electronic supplementary material, S1 for detailed methods and S2 for elicitation spreadsheet sent to experts). We elicited expert knowledge to estimate the consequence score, conducting surveys among experts (local knowledge holders, scientists, and managers) for each of the ecosystem components through emails, online conferences, and phone interviews. During the elicitations, we provided a spreadsheet detailing pressure-risk variable combinations (electronic supplementary material, S2) and asked experts to score or qualify the consequence variable for each species-pressure pair. Following [29], each of the four risk variables (temporal scale, spatial scale, load, and consequence) was assigned an uncertainty term (electronic supplementary material, table S3). Monte Carlo simulation was used to incorporate this uncertainty explicitly into the calculation of risk (see the electronic supplementary material, S1 for full calculation of uncertainty).

#### (i) Direct risk

The cumulative direct risk of pressures to each ecosystem component was calculated by summing the total risk score produced for each ecosystem component across all its pressures, within each iteration of the Monte Carlo simulation using the statistical platform R (v. 4.0.5, R Core Team 2016). Direct cumulative risk *Crisk* to each ecosystem component *j* was calculated by summing risk across all pressures as follows:

$$Crisk_{j}=\underset{i}{\sum }risk_{ij}.$$

#### (ii) Indirect risk

We used our conceptual model to inform the direction of indirect (bottom-up) pathways by which risk from pressures might transfer between ecosystem components (figure 1). The ‘comprehensive’ cumulative risk (CC*risk_j_*) to each ecosystem component *j* was calculated as:

$$\mathrm{CC}risk_{j}=Crisk_{j}+\underset{e}{\sum }p\mathrm{CC}riske\;,$$

which included 100% of its direct cumulative risk (*Crisk_j_*), plus the sum of the proportion (*p*) of the risk to each of its supporting ecosystem components (*e*, indirect risk), including prey species or habitat (if applicable) [29]. We included two-way relationships between ecosystem components when each component was dependent on the other, e.g. bears dependent on berries for food, and berries dependent on bears for dispersal [34,35]. The model evaluates risk to low trophic species first, and then upper trophic species, so that primary (immediate prey) and secondary (components that have bottom-up links to prey) indirect linkages are included for upper trophic species.

Previous analyses applied a conservative, well-accepted energy transfer relationship of 10% [29,36] to the risk scores to reflect relationships between species in the network, based on ecological efficiency and energy transfer. Our baseline assessment uses this energy transfer value for *p* for all interaction pathways, based on [29]. As a full example, comprehensive cumulative risk to the ecosystem component ‘bears’ is estimated by the direct risk to bears plus 10% of the risk to its prey items (relationships demonstrated in figure 1): salmon, berries, clams, herring, forest. In turn, the comprehensive cumulative risk to salmon was calculated using its linkage framework, including the biogenic habitats kelp and eelgrass. Although previous analyses assume a 100% transfer risk from salmon to resident orca [29] based on their obligate dependency on salmon prey across the BC coast, we here apply a risk transfer of 10%, assuming that these species are moving in and out of the region and are feeding in other areas of BC [37].

A homogeneous transfer efficiency assumes implicitly that the importance of each prey item is uniform in its contribution to a predators' diet. The contribution of multiple different prey to a predator's diet is rarely uniform, however, and assimilation efficiencies can vary among trophic groups [38]. We conducted a sensitivity analysis where we improve upon [29] by weighting the risk-transfer relationship or proportion (*p*) according to available information in the literature on production and assimilation efficiencies for marine mammals and anadromous fishes, and habitat use for benthic habitats and fauna (electronic supplementary material, S1, tables S4 and S5). For the purposes of this analysis, we assumed all prey were accounted for, but we acknowledge that there are probably other food sources in both the marine and land food web component that have not been included. In case the risk to a known prey species was not scored during the case study, the risk to the trophic groups was estimated by adding 10% of the risk to the ecosystem component representative (e.g. for any species consuming a pelagic forage fish other than Pacific herring, we added to its cumulative risk 10% of the cumulative risk to Pacific herring; all representatives for trophic groups are described in table 1). For those that there is no information on relationships, we kept the accepted energy transfer relationship of 10%.

#### (iii) Scenarios of change

We examined the magnitude of potential change in cumulative risk for each ecosystem component when we included future climate change (hereafter ‘future’ scenario). In addition to the pressures from human activities, we included seven new pressures: temperature change, sea-level rise, insect and disease defoliation, ocean acidification, thermal sensitivity—freshwater, temperature extremes—glacial melt and habitat shifting and alteration (electronic supplementary material, table S1). These pressures were chosen by the experts based on their knowledge of the region and existing evidence of climate change impacts. The final future scores were compared to the original.

We also examine the magnitude of change in cumulative risk for each ecosystem component as it propagates through the food web when we manage risk from local human activities (hereafter ‘manage’ scenario) for foundation prey species. We selected the pressure with the highest summed risk for salmon and herring, (excluding climate drivers, which cannot be managed locally, biological processes and long-term pressures), and chose the associated activity for management. To simulate management actions, we manually reduced the temporal exposure score for all pressures listed in that activity (see the electronic supplementary material, table S1 for activities in the realm associated with that species) by one level (e.g. if the exposure score was 4, then it was reduced to 3), and evaluated the final risk compared to the original:

$$\mathrm{CC}riskj\_manage=Criskj\_manage+\underset{s}{\sum }p\mathrm{CC}risks\_manage\;.$$

We chose to reduce temporal scores only to simulate a scenario where human activities were restricted seasonally, for example, a change in timing of the fishery to avoid salmon spawning runs, rather than spatially, as in many instances the spatial score was already low (i.e. less than or equal to 10–100 kms), and so reducing the spatial scale of those pressures was not logical. Mathematically, however, the calculation would be similar if we manually reduced the spatial exposure score, or the load score, by one value. We only reduced temporal scores for those pressures where a lower risk category was available. We applied the same uncertainty calculations as for the baseline model. We conducted two management scenarios—one for land pressures, and one for marine pressures.

#### (iv) Land-sea models

We developed multiple models to explore how management and assessment of risk within a single-realm (e.g. marine or land-based) might differ from integrated decision making that considers land-sea connections. First, we developed a fully integrated land-sea model (cross-realm), which includes trophic interactions between species across realms, and all relevant pressures. We then developed a single-realm ‘land-only’ model, that excludes food web links to marine species and pressures, and includes only those pressures driven by land activities, and developed a single-realm ‘marine-only’ model, that excludes food web links to land species and excludes land activities. This resulted in three realm models (cross-realm, land, and marine), and three scenarios (‘baseline’, ‘future’, and ‘manage’) for each model. Freshwater pressures were included in the land model, as they were directly associated with land activities (forestry, mining), and salmon linkages were included in all models. We calculated risk statistics (median and error (10% and 90% percentiles)) for direct, indirect, and cumulative risk across ecosystem components for each model and scenario and compare between models and scenarios. We also compare summed and average statistics between land-only species, and marine-only species (table 1).

We used the nonparametric Spearman rank correlation test to examine the relationship between the median comprehensive, direct and indirect risks for each ecosystem component. We also built three generalized linear models (GLMs) in R to understand if cumulative risk was a function of species' connectedness, i.e. species with more linkages have higher risk than species with fewer linkages. The predictor variable was the number of prey and habitat linkages for each species, and dependent variables were compared between the comprehensive and indirect risk score. We then ran alternative models where we include a binary parameter (0,1) indicating whether the ecosystem component relies on foundation species (salmon or herring) as a primary food source. We compared three model sets: cross-realm, marine-only and land-only. Model fit for each model set (cross-realm, land, marine) was evaluated by residual diagnostics and the choice of the final model was guided by Akaike's Information Criterion (AIC).

## 3. Results

### (a) Baseline cross-realm model

**Figure 2.**
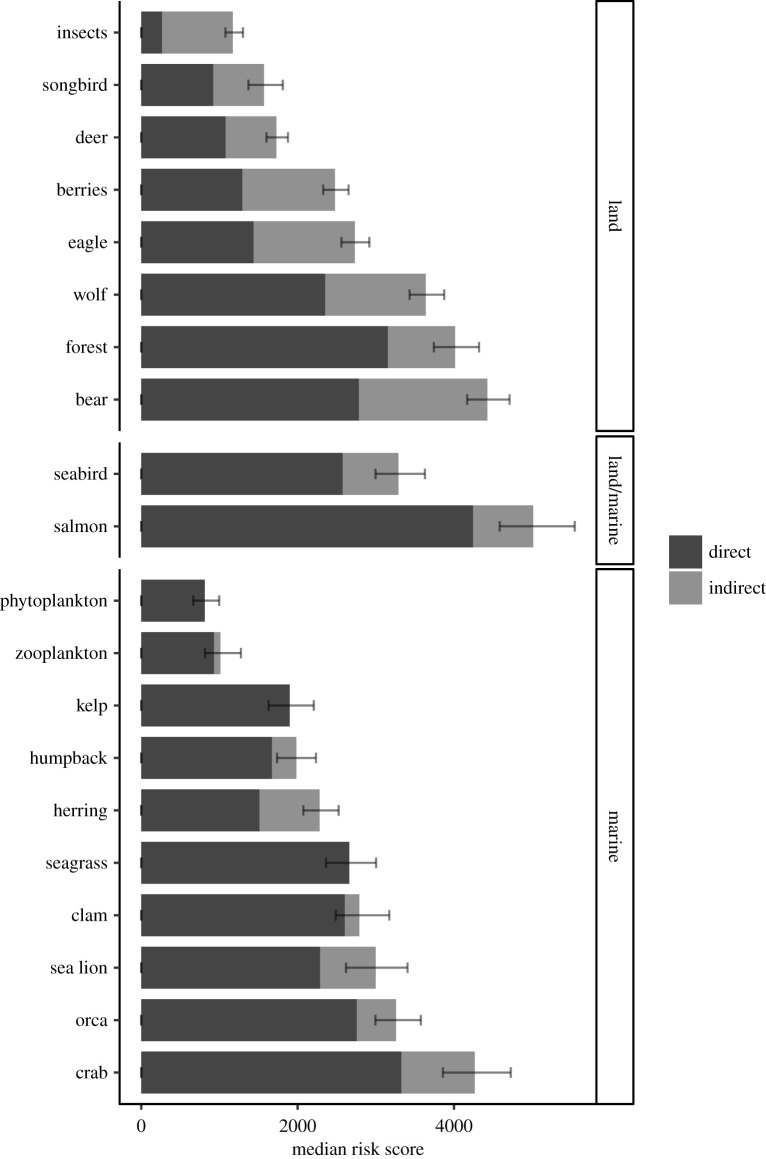
Median cumulative risk for each ecosystem component for the cross-realm model, including 10th/90th quantile error bars for the comprehensive score, sorted by realm.

Comprehensive risk scores for the cross-realm baseline model that included land and marine linkages were highest for salmon, followed by bears, Dungeness crab, old growth forest and wolves (figure 2). The highest direct risk from pressures alone were for salmon again, followed by crab and forest (figure 2). The top three pressure scores summed across all ecosystem components were for land activities (forestry and land-use change; electronic supplementary material, table S6 and figure S1). The highest direct score (pressures only) summed across all ecosystem components was for logging (long-term loss of forest structure), which was more than three times greater than the highest marine pressure score (tourism: marine vessel oil and contamination).

On average, the comprehensive risk was 12% higher for land-only components than marine-only components. Ecosystem components supported by foundation species, i.e. salmon and herring, had higher comprehensive risk scores (46% and 62% greater on average, respectively) than ecosystem components not supported by these species. The highest indirect risk for the cross-realm model was for land-associated ecosystem components, primarily bears (37% of comprehensive risk), followed by eagles, wolves and berries.

The comprehensive risk was on average 68% greater than direct risk for land-only ecosystem components, versus 15% for marine-only, despite there being fewer land pressures. The greatest change between direct scores and comprehensive scores was for insects, whose comprehensive score was four times greater than the direct score, largely owing to a very low direct risk (less than 300, figure 2). Large increases in the comprehensive risk owing to the addition of indirect effects were also seen for berries and eagles, for which indirect risks made up 47% of their comprehensive score (figure 2).

#### (i) Changes to risk from using single-realm models

**Figure 3.**
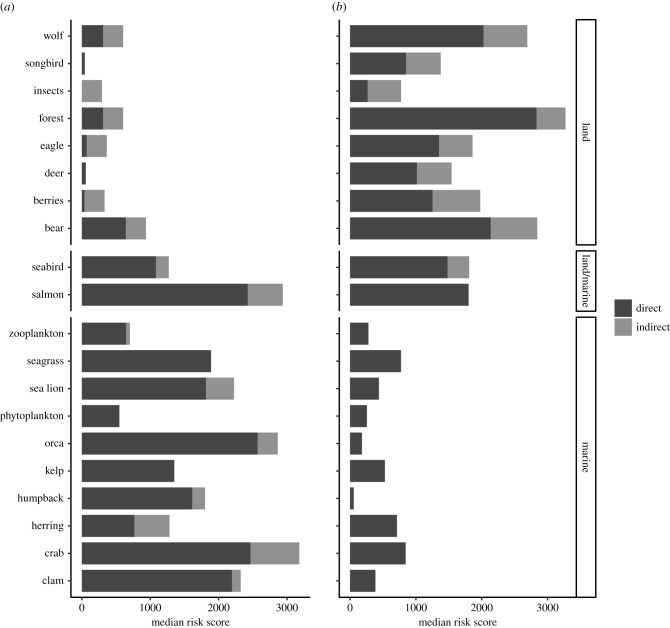
Median cumulative risk for each ecosystem component, for the baseline marine-only model (left) and the land-only model (right), identifying direct and indirect components. Both models exclude cross-realm indirect linkages, and includes only those pressures associated with marine or land activities respectively. Salmon linkages were included in both models.

There were large reductions in the comprehensive risk for marine-associated ecosystem components when we removed land prey, habitat linkages and pressures in the ‘marine-only’ model, especially for marbled murrelets (greater than 60% of total risk lost), herring and salmon (44% and 41% of total risk lost, respectively) (figure 3). Indirect risks for the marine-only model were highest for crabs, followed by herring and salmon (figure 3). In the ‘land-only’ model, more than 30% of the comprehensive score was lost for bears, eagles and insects (figure 3). Indirect risks, mediated by land prey and habitat linkages, were highest for bears, wolves, and berries in this model. Average scores for the land-only model were 4% higher on average for indirect effects, and 7% lower for direct effects, than for the marine-only model and associated ecosystem components.

Ranking all ecosystem components by cumulative score showed substantial differences between cross-realm and single-realm models. Salmon and herring were 7th and 13th most at-risk, respectively, from land/freshwater pressures alone, and moved to 2nd and 9th most at-risk from marine pressures (electronic supplementary material, table S7). Crab, ranked most at-risk from marine pressures, moved to 10th position in the land-only model (electronic supplementary material, table S7). By contrast, forest, bears and wolves were the top three most at-risk in the land-only assessment, and moved positions only slightly to 4th, 2nd and 5th most at-risk respectively when land-sea linkages were considered (electronic supplementary material, table S7).

#### (ii) Statistical models and sensitivity analyses

**Figure 4.**
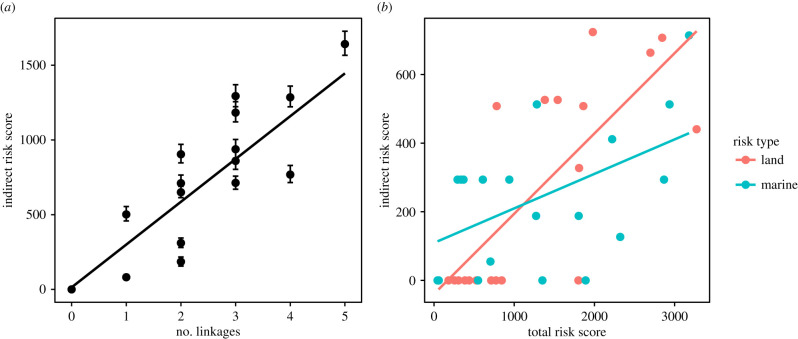
(*a*) Comparison of the number of prey and habitat linkages and indirect risk for the cross-realm model (black), and fitted linear trend line; (*b*) comparison of median comprehensive risk and indirect risk for each ecosystem component, for the marine-only model (blue) and the land-only model (red), for the baseline scenario, with fitted linear trend lines. (Online version in colour.)

There was a significant strong positive correlation for the cross-realm model between the number of linkages (prey and habitat inputs) and both comprehensive (Spearman rank, cor = 0.66, d.f. = 18, *p* = 0.002; figure 4*a*) and indirect risk (Spearman rank, cor = 0.86, d.f. = 18, *p* < 0.001; figure 4*a*). There were significant positive correlations between linkages and indirect risk for the land-only model (Spearman rank, cor = 0.71, d.f. = 18, *p* < 0.001), and marine-only model (Spearman rank, cor = 0.70, d.f. = 18, *p* < 0.001; figure 4*b*). Comparison of GLMs revealed the best-fit models (lowest AICc) for each set were those that included linkages and foundation species (electronic supplementary material, table S8).

Modifying the risk transfer value for each ecosystem component (electronic supplementary material, table S5) resulted in a 15% reduction in comprehensive risk, but no change to the top three species' rankings (electronic supplementary material, figure S2). Land-associated ecosystem components had the greatest reductions in risk from modifying the risk transfer value, on average 3% more than marine species. For marine-only species, the greatest reductions in risk were for seagrass and kelp, whereas humpback whales and herring increased in comprehensive risk by less than 1%.

### (b) Future scenario

**Figure 5.**
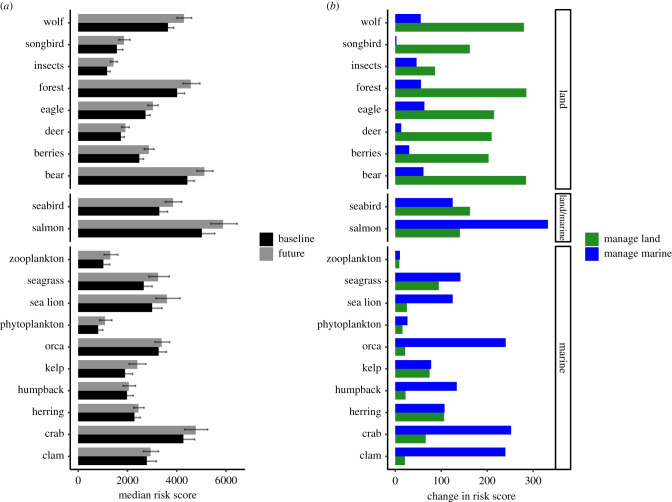
(*a*) Median comprehensive risk for each ecosystem component, for baseline, versus future management, for the cross-realm model; (*b*) reduction in total risk from reducing forestry pressures in the manage land scenario (green) versus reducing fisheries pressures in the manage marine scenario (blue). (Online version in colour.)

The inclusion of additional climate change pressures increased the summed comprehensive risk for all ecosystem components as expected, with a 13% increase in the cross-realm model. Salmon, crab and bear retained the highest comprehensive risks for current and future scenarios (figure 5*a*). The greatest relative increases in comprehensive risk under climate change were for low trophic marine components (phytoplankton, zooplankton, kelp; greater than 25% increase) and land components (insects; greater than 40% increase) (figure 5*a*). The smallest relative changes were for humpback whales, orca and clams, which increased in comprehensive risk by less than 5% in the futures scenario (figure 5*a*). Forestry retained the highest combined pressure score (summed across all ecosystem components), followed by temperature change from climate activity.

Including future climate pressures into the single realm models increased summed comprehensive risk across all ecosystem components by 17% for the marine-only assessment and 14% for the land-only assessment (figure 5*a*). In the marine-only future model, the largest increases in indirect scores from the baseline model were for zooplankton (50% increase), followed by clam (45% increase), herring and salmon (30% increase). In the land-only future model, the largest increases in indirect scores from the baseline model were for forest and berries (19% and 15% increase respectively). Increased summed risk in the land-only future model was driven by increased indirect risks for certain ecosystem components (forest, bears and wolves), with the average increase in direct risk only 1%.

### (c) Manage scenarios

The pressures with the highest cumulative risk for salmon and herring were associated with forestry activity for land (pollution from forestry effluents (i.e. sedimentation), and estuarine/inshore habitat disturbance respectively), with commercial fisheries ranking the highest for marine activities (ranked 5th across all pressures for salmon).

When we reduced temporal exposure scores for either forestry- or fisheries-related pressures to simulate a land management and a marine management scenario (electronic supplementary material, table S9; for all original scores see S3), marine management resulted in the greatest risk reduction for a single ecosystem component, salmon (5.7% decrease; figure 5*b*). Substantial reductions in scores for the marine management scenario were also seen for crab, orca and clam (figure 5*b*). The greatest risk reductions in the land management scenario were for old growth forest, bears and wolves (figure 5*b*), driven by reduced direct (6–7%) and indirect scores (4–5%). Of the marine-associated ecosystem components, the greatest decreases in risk score from the current to the managed land scenario were for marbled murrelets, salmon and herring (figure 5*b*). The total reduction in risk summed across all ecosystem components for land management was 14% greater than that for marine management.

## 4. Discussion

The rapid approach to risk assessment provided here helps decision-makers quickly identify risks from human activities at an ecosystem level and to species, to pinpoint where management might be most effective, and garner information on how to best support overall resilience of the system. Ecosystem assessment tools that account for both indirect and direct linkages across the whole system ensure that critical changes can be both predicted and evaluated prior to irreversible ecological degradation and collapse [25]. We illustrate that ignoring indirect interactions underestimates cumulative risks to species of human activities by up to 68% for land species, versus 15% for marine species. Our approach also highlights the importance of considering pressures across both land and marine realms in coastal ecosystems, with the predicted risk of human activities increasing when land-sea linkages were considered by up to 44% for marine species (e.g. herring and salmon) and 37% for land species (e.g. bears and eagles). Our results show that large reductions in risk across the ecosystem can be achieved by managing key threats to foundation species such as salmon and herring. We found direct risks associated with marine pressures were highest across species for salmon, with management of fisheries pressures resulting in the greatest risk reduction for both for salmon and their key marine predators (orca and sea lion) versus managing land pressures (forestry, figure 5*b*), suggesting salmon management cannot be done solely from a land (i.e. freshwater) perspective.

More broadly, despite recognition of the need for whole-ecosystem approaches to risk assessment, many marine evaluations still focus on single activities and/or single species (e.g. fisheries bycatch) [39–41]. Our study shows that risk assessments which account for only marine or land pressures can result in substantial differences in perceptions of cumulative risk to species in coastal ecosystems; some species’ cumulative risks were underestimated by single-realm assessments that ignored cross-realm linkages (e.g. crab and salmon, electronic supplementary material, table S7). Consideration of cross-realm linkages with prey or habitats made a greater difference to the cumulative risk of land-associated species and habitats compared with marine components—for land-associated species, ignoring marine lower trophic linkages and pressures affecting marine ecosystem components underestimates on average more than 40% of total cumulative risk to the ecosystem as a whole. Although this indicates greater negative consequences for these land components, it also may reflect a difference in the understanding (literature and elicited responses) of how pressures manifest as impacts in the marine environment compared to the terrestrial environment, or even of the differences in our understanding of food webs. For species that are dependent on both realms (e.g. marbled murrelets, salmon), up to two-thirds of the total cumulative risk might be underestimated if marine linkages are ignored (electronic supplementary material, table S7). We show that cross-realm species are more vulnerable because they are not only exposed to multiple pressures but they also usually have more links with other ecosystem components through their prey, multiple cross-realm predators, and use of multiple habitats ([42], table 1, figure 1). These results support calls for land-sea assessments to be done simultaneously rather than independently as is more generally done.

The need to consider indirect effects from land activities on linked marine ecosystems for effective management is increasingly recognized [22,43,44], particularly in marine spatial planning (e.g. [45,46]). However, research to inform management of threatened species that recognizes and explicitly includes indirect cross-realm predator-prey linkages are few. Our findings highlight flow-on effects of human activities up the food web, not only for high-trophic level predators like bears and wolves, but also lower trophic groups such as salmon-dependent riparian zones and forests, insects, birds, and berries. Importantly, we show predators with links to salmon and herring are disproportionally affected by pressures in the region when indirect risks are considered (electronic supplementary material, table S8), with average total risks for these species more than 60% greater than those for low trophic species, or for those not supported by these foundation species. We also found greater importance of including indirect risk transfer through bottom-up processes in our land-only and cross-realm models (figure 4), reinforcing the need for consideration of ecosystem-based thinking that explicitly considers predator-prey interactions when evaluating threats to land species. Our approach can also be used to pinpoint which pressures exert the greatest single-realm and cross-realm impacts. At an ecosystem scale, logging activities currently pose the greatest risk to land and marine species and habitats in the Central Coast study region (electronic supplementary material, table S6). Reducing the temporal frequency of forestry pressures resulted in greater whole-ecosystem risk reductions compared with reducing commercial and recreational marine fishing pressures (figure 5*b*). This supports previous research that suggests the best way to manage this system is through integrated ecosystem-based management [19].

We make several assumptions with the models used in this assessment. The model does not consider interactions between pressures. Particularly for understanding the risks associated with future climate change, this information may be needed to understand how, when, and where local pressures should be managed [47]. The assumption of additive cumulative impacts in the model is somewhat simplistic and may fail to reflect nonlinear interactions [48,49], particularly to large-scale climate variability. Although we compiled a comprehensive list of pressures formed from expert elicitation and literature review, we aggregated some in the marine realm so that the number of marine pressures was more comparable to land and freshwater pressures (e.g. fisheries was previously disaggregated by gear [29], here we combine these under one pressure). We also chose only a subset of potential future pressures, and alternative scenarios could be examined in future research. Similarly, although we explore the magnitude of direct effects from pressures versus bottom-up effects mediated by prey and habitat linkages, we do not evaluate indirect effects associated with changing predator abundances. For example, if a predator declines and that reduces prey mortality, would that relieve some of the consequences of human pressures? Such effects could be explored using a dynamic ecosystem modelling approach [28], however such methods require substantial data and resources.

Managers need tools to understand cumulative risks to species from human activities and to bolster ecosystem-based management. Salmon- and herring-supported ecosystems are highly complex social-ecological systems [50]. The complexity of linkages and indirect impacts of activities on different ecosystem components complicates management and research, as a management action targeted towards a single species can have flow-on effects to the whole ecosystem [51]. Here, we demonstrated a rapid assessment approach to help managers prioritize key stressors to address in a complex interconnected land-sea system. By identifying where pressures have greatest impacts (i.e. land versus marine; figures 2 and 3), and which pressures have the greatest cumulative effect on the whole ecosystem (electronic supplementary material, table S6), we provide critical information necessary for prioritizing management actions and highlight trade-offs between managing land-based versus marine-based activities. Importantly, our findings reiterate the need for consideration of cross-realm linkages for effectively managing coastal foundation species at ecosystem levels.

## Data Availability

The datasets and code supporting this article have been uploaded as part of the electronic supplementary material. The expert elicitation spreadsheet is electronic supplementary material S2. Scores for all risk metrics can be found in S3, code for the models is found S4 [52].
